# Supplementary material for: Protein folding while chaperone bound is dependent on weak interactions
Source: Nat Commun. 2019 Oct 23;10:4833. doi: 10.1038/s41467-019-12774-6 (PMC6811625; doi:10.1038/s41467-019-12774-6)
Supplement: Supplementary file 1 — Supplementary Information [file 41467_2019_12774_MOESM1_ESM.pdf]

Supplementary Information

Protein folding while chaperone bound is dependent on weak interactions

Kevin Wu *et al*.

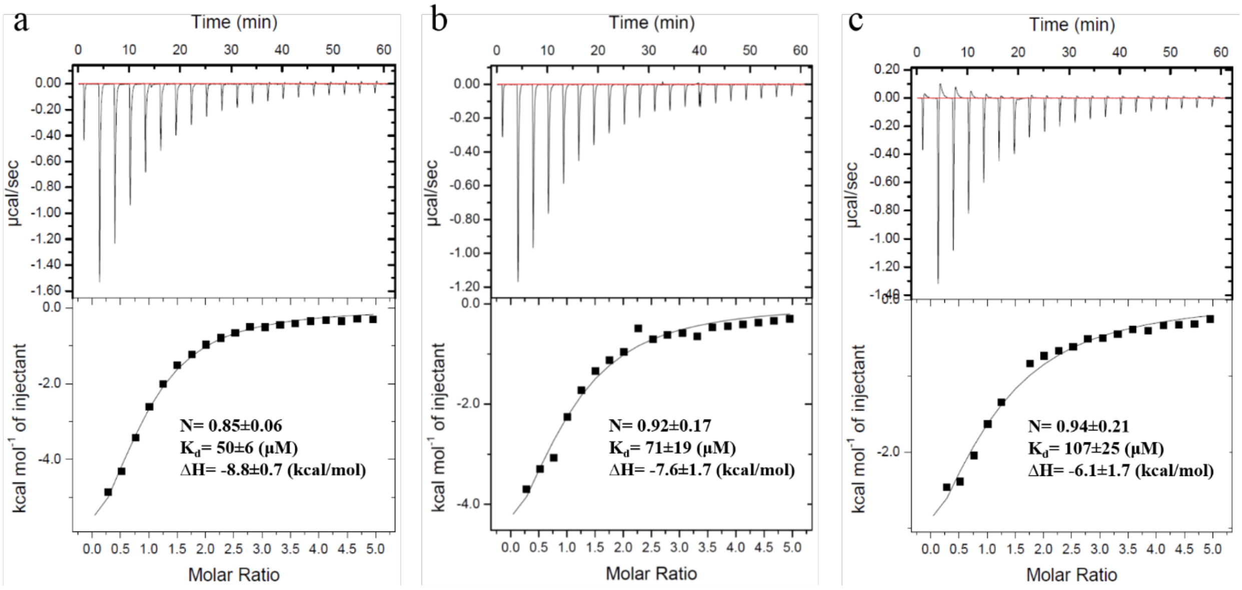

Supplementary Figure 1 ITC analysis for SH3 binding to wild-type Spy and variants. All the experiments were conducted at 25 °C. 2.4 mM SH3 in the syringe was titrated into 100 µM wild-type Spy **(a)**, SpyH96L **(b)** and SpyQ100L **(c)** in HN buffer containing 0.83 M urea, which was consistent with the buffer used in our stopped-flow experiments. The best fit of the data to a one-site model is shown with the black line in the bottom panels.

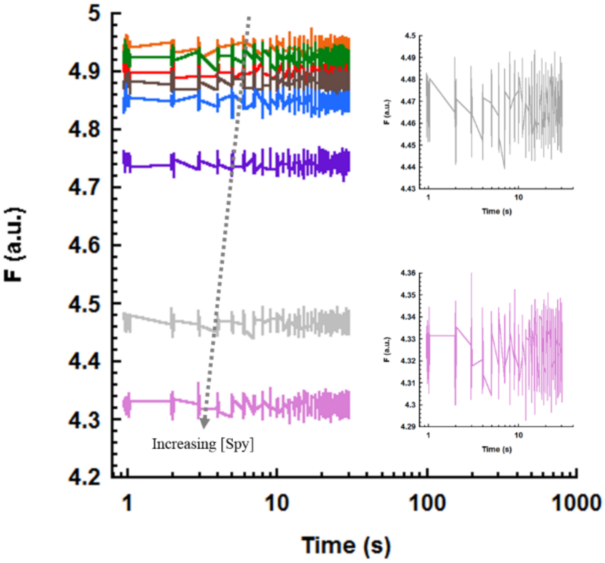

Supplementary Figure 2 Tryptophan fluorescence traces for native SH3 binding to wild type Spy on a logarithmic timescale. Traces of SH3 binding to Spy in the presence of 160.7 µM Spy and 228.9 µM Spy, colored in grey and pink, respectively, are shown individually in the insets on the right.

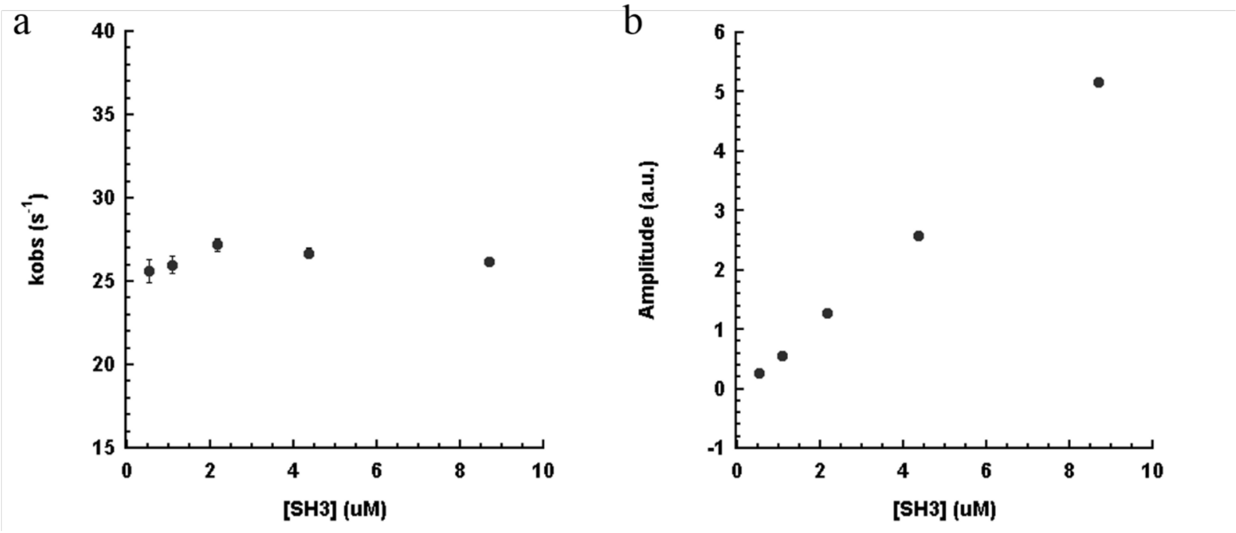

Supplementary Figure 3 The refolding kinetics of SH3 at a range of concentrations. **a** The observed rate constants for the SH3 refolding at various concentrations (from 0.5 μM to 8.7 μM) are very similar. **b** The amplitude of the SH3 refolding traces increase linearly with the SH3 concentrations. Errors were derived from the fitting standard errors for the traces that represent the avrage of 10-15 individual traces.

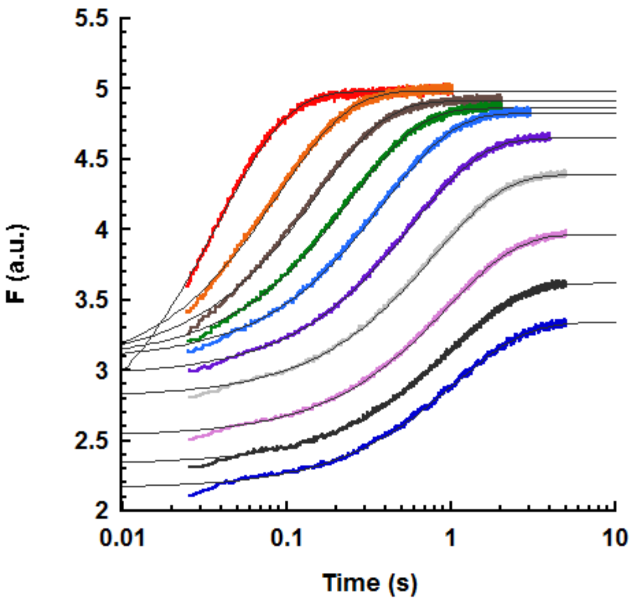

Supplementary Figure 4 All the refolding traces in the absence and in the presence of Spy can be well described by single-exponential.

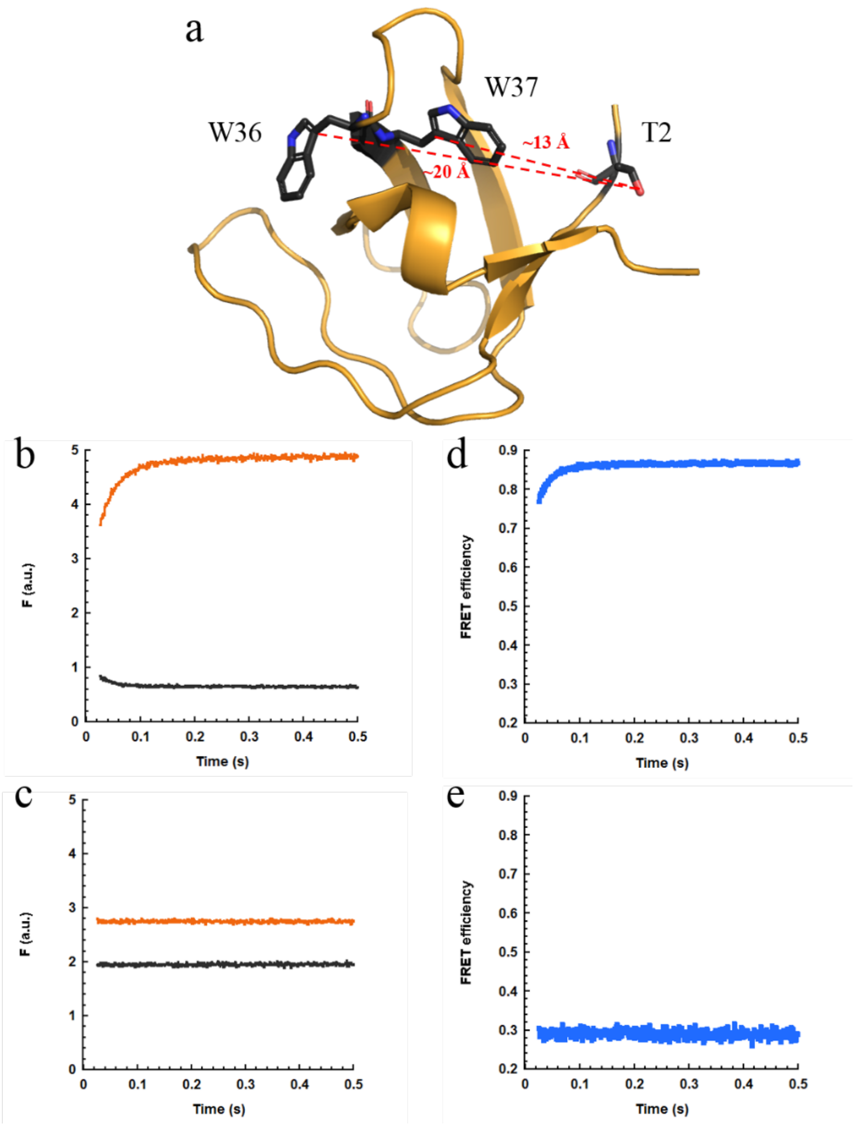

Supplementary Figure 5 SH3 refolding kinetics monitored by stopped-flow FRET. **a** Structure of Fyn SH3. Trp36, Trp37 and Thr2 are shown as black sticks. **b** The changes in fluorescnece intensity of unlabeled and TNB-labeled SH3, shown in orange and black, respectively, during refolding. Both kinetic traces can be fit by two-exponentials. The kobs for the fast and slow phase in the refolding traces of unlabeled SH3 are 34±1 and 6.4±0.6 s^{-1}, respecitvely. The kobs for the fast and slow phase in the refolding trace of TNB-labeled SH3 are 42±3 and 4±2 s^{-1}, respecitvely. **c** The fluorescnece intensity of unlabeled and TNB-labeled SH3, shown in orange and black, respectively, in 9.5 M urea buffer. **d** The change in FRET efficiency during refolding was calculated from the corresponding fluorescence intensity of unlabeled and TNB-labeled SH3, as shown in (**b**) using Eq. 1. **e** The FRET efficiency for SH3 in 9.5 M urea was calculated from the corresponding fluorescence intensity of unlabeled and TNB-labeled SH3 as shown in (**c**).

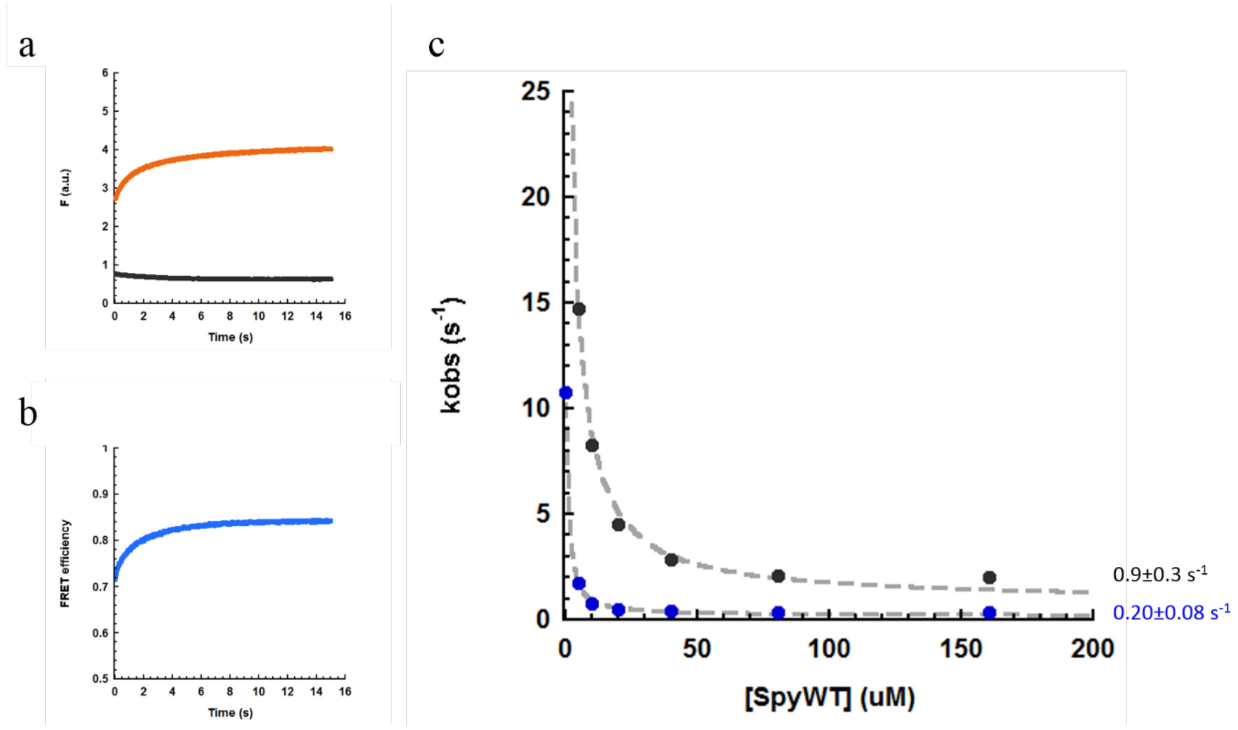

Supplementary Figure 6 Stopped-flow refolding kinetics of SH3 in the presence of Spy probed by FRET. **a** The changes in fluorescence intensity of 4.8 μM of unlabeled and TNB-labeled SH3, shown in orange and black, respectively, in the presence of 160.7 μM of Spy during folding. **b** The changes in FRET efficiency were calculated using Eq. 1. All the traces for SH3 refolding can be fit by two-exponentials. **c** Plot of kobs for the fast and slow phase in SH3 refolding as a function of Spy concentration.

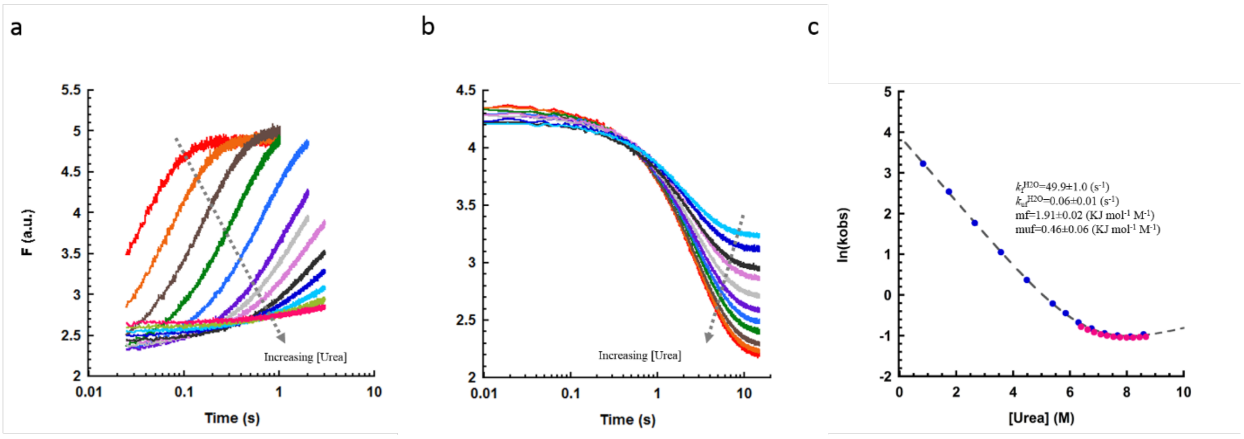

Supplementary Figure 7 Urea-dependent SH3 folding and unfolding. SH3 refolding **(a)** and unfolding **(b)** was initiated by 11.5-fold dilution of SH3 in 9.5 M urea and 0 M urea, respectively, with HN buffer containing various concentrations of urea. **(c)** k_{obs} was obtained by fitting the traces to a single exponential and plotted as a function of the urea concentration. The data points from the folding experiments are colored in blue, and those from the unfolding experiments are colored in pink. The combined data set was fitted to an equation describing a two-state transition: $\ln(k_{obs}) = \ln(k_f^{H2O}e^{(-mf/RT)[urea]} + k_{uf}^{H2O}e^{(muf/RT)[urea]})$, where k_{obs} is the observed rate constant, k_f^{H2O} and k_{uf}^{H2O} are the microscopic rate constant in the absence of urea for the refolding and unfolding of SH3, respectively, mf and muf define the denaturant dependence of k_f^{H2O} and k_{uf}^{H2O}, and R and T are the gas constant and 298 K, respectively.

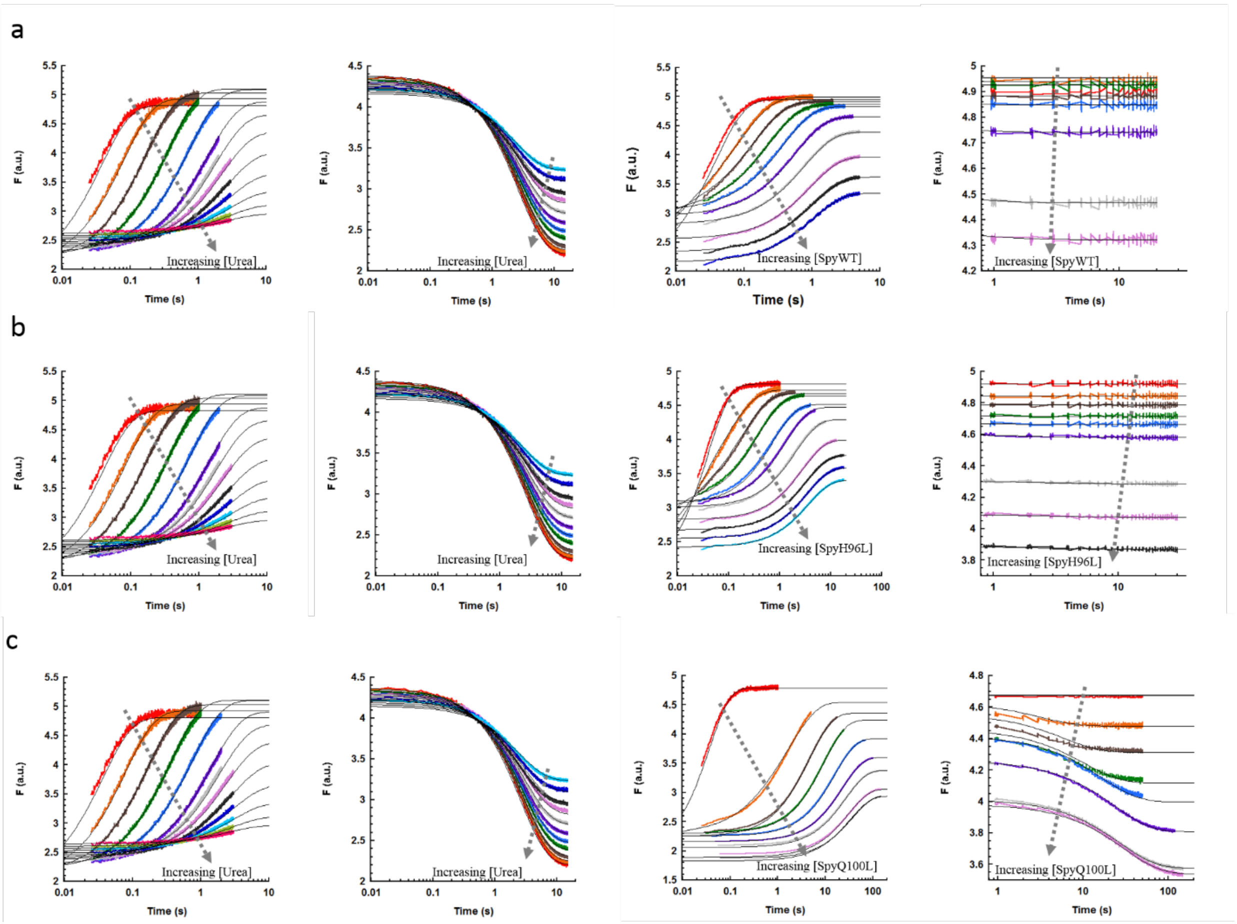

Supplementary Figure 8 Simultaneously fitting all kinetic data to the mechanism that allows the folding of SH3 while bound to Spy **(a)**, SpyH96L **(b)** and SpyQ100L **(c)**. From left to right are the fluorescence traces for the folding kinetics of SH3 at various concentrations of urea, fluorescence traces for the unfolding kinetics of SH3 at various concentrations of urea, fluorescence traces for the folding kinetics of SH3 in the presence of different concentrations of Spy or its variants in 0.83 M urea, and fluorescence traces for the binding kinetics of SH3 with different concentrations of Spy in 0.83 M urea. All the experiments were conducted in 40 mM HEPES (pH 7.5) and 50 mM NaCl at 25 °C. All the kinetics parameters obtained from the fitting results can be found in Table 1.

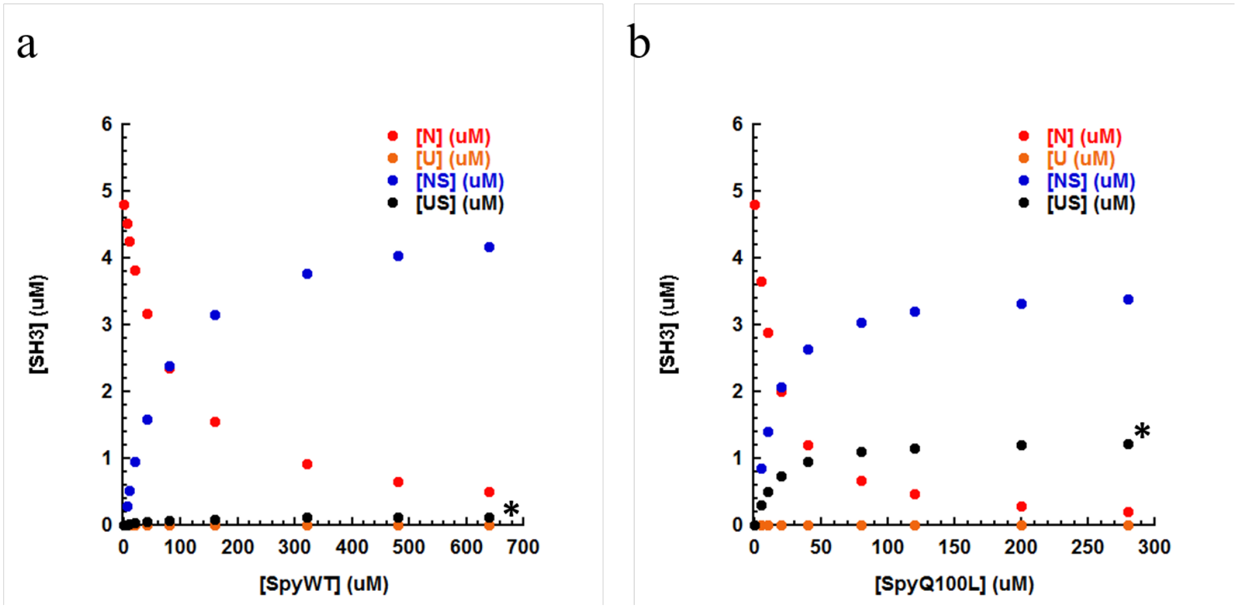

Supplementary Figure 9 The concentrations of SH3 in the presence of various concentrations of wild-type Spy **(a)**, and SpyQ100L **(b)** are simulated from the corresponding kinetic models. The concentrations of Spy-bound unfolded SH3 in the presence of Spy and SpyQ100L were marked as asterisk.

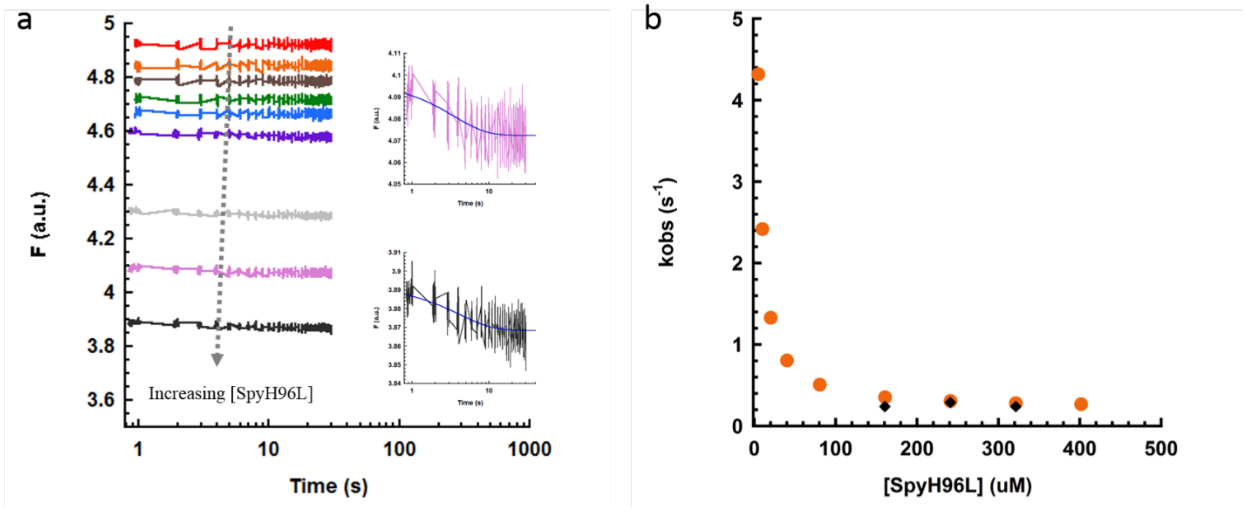

Supplementary Figure 10 Fluorescence traces for SH3 binding to SpyH96L. **a** The fluorescence amplitude of the slow phase is tiny at low SpyH96L concentrations, making it difficult to obtain accurate k_{obs} values at these concentrations (below 100 µM). At high concentrations of SpyH96L (160.7 µM in grey, 241.0 µM in pink, and 321.4 µM in black), the k_{obs} is 0.24 ± 0.02 s^{-1}, 0.29 ± 0.03 s^{-1}, and 0.24 ± 0.02 s^{-}1, respectively. **b** An overlay of the k_{obs} measured in the binding experiment (black diamond) with the k_{obs} determined from the SH3 refolding kinetics (orange circle) as a function of SpyH96L concentrations (Fig. 4c).

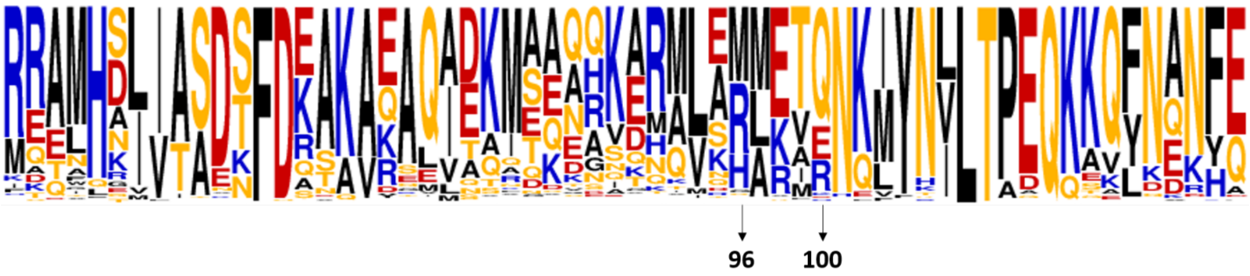

Supplementary Figure 11 Sequence alignment of Spy sequences obtained from PSI-BLAST searches. Only the small portion of alignment including positions 96 and 100 is shown. Numbers are based on the mature *E. coli* Spy sequence.

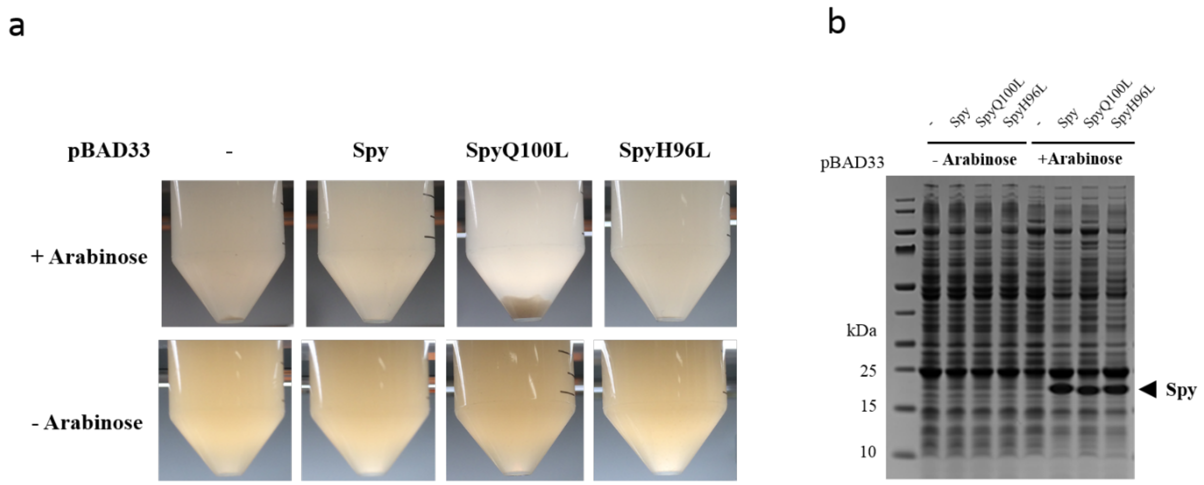

Supplementary Figure 12 Expression and clumping phenotype of Spy and its variants. **a** Expression of Spy Q100L in the *E. coli.* cytoplasm induces the formation of cell clumps. Cells cultured in M9 minimal medium for 35 h (see Methods) were transferred to a 50 ml conical tube and spun down at 1000 x g for 10 s to precipitate cell clumps. **b** Expression level of Spy and its variants in M9 minimal medium-cultured O.D. 1.0 cells examined by SDS-PAGE and subsequent Coomassie staining.

Supplementary Table 1

The stability of native SH3 and Im7

	k_f (s^{-1})	k_{uf} (s^{-1})	ΔG_{UN} (KJ mol^{-1})		
SH3	26.7±0.1	0.0290±0.0001	-16.9±0.1		
	k_{UI} (s^{-1})	k_{IU} (s^{-1})	k_{IN} (s^{-1})	k_{NI} (s^{-1})	ΔG_{UN} (KJ mol^{-1})
Im7	1610±210	1940±180	286±23	0.99±0.01	-13±2

Rate constants for SH3 folding and unfolding were obtained from the global fitting analysis shown in Table 1. The change in the free energy of unfolding, ΔG_{UN}, was calculated from the equation $\Delta G_{UN} = RT \ln (k_f / k_{uf})$, where k_f is the folding rate for SH3 at 0.83 M urea, k_{uf} is the unfolding rate for SH3 at 0.83 M urea, R is the gas constant, and T is 298 K. Rate constants for Im7 folding and unfolding were obtained from Table 1 in our previous study[1]. ΔG_{UN} for Im7 three-state folding mechanism was calculated from the equation $-RT \ln [K_{UI} (k_{IN} / k_{NI})]$[2], where K_{UI} is the equilibrium constant between unfolded and intermediate states, k_{IN} and k_{NI} are the folding and unfolding rate constant of the transition between intermediate and native state, respectively.

Supplementary Table 2

The parameters of FRET.

	J*10^{-13} (M^{-1} cm^{-1} nm^4)	Q_D	n	Ro (Å)
Native state	7.3	0.18	1.33	25.0
Unfolded state	7.4	0.13	1.41	22.9

J, overlay integral of donor emission and acceptor absorbance;Q_D,quantum yield of the donor fluorescence; n, refractive index of solvent; R_0, Förster distance.

Supplementary References

1 Stull, F., Koldewey, P., Humes, J. R., Radford, S. E. & Bardwell, J. C. A. Substrate protein folds while it is bound to the ATP-independent chaperone Spy. *Nat Struct Mol Biol* **23**, 53-58, doi:10.1038/nsmb.3133 (2016).

2 Ferguson, N., Capaldi, A. P., James, R., Kleanthous, C. & Radford, S. E. Rapid folding with and without populated intermediates in the homologous four-helix proteins Im7 and Im9. *Journal of Molecular Biology* **286**, 1597-1608 (1999).
